# Methods to Design and Synthesize Antibody-Drug Conjugates (ADCs)

**DOI:** 10.3390/ijms17020194

**Published:** 2016-02-02

**Authors:** Houzong Yao, Feng Jiang, Aiping Lu, Ge Zhang

**Affiliations:** 1 Institute for Advancing Translational Medicine in Bone & Joint Diseases, School of Chinese Medicine, Hong Kong Baptist University, Hong Kong, China; yaohouzong@163.com (H.Y.); jiangfeng@nbu.edu.cn (F.J.); 2 Faculty of Materials Science and Chemical Engineering, the State Key Laboratory Base of Novel Functional Materials and Preparation Science, Ningbo University, Ningbo 315211, Zhejiang, China

**Keywords:** antibody-drug conjugates (ADCs), targeted therapy, monoclonal antibodies (mAbs), drugs, linkers

## Abstract

Antibody-drug conjugates (ADCs) have become a promising targeted therapy strategy that combines the specificity, favorable pharmacokinetics and biodistributions of antibodies with the destructive potential of highly potent drugs. One of the biggest challenges in the development of ADCs is the application of suitable linkers for conjugating drugs to antibodies. Recently, the design and synthesis of linkers are making great progress. In this review, we present the methods that are currently used to synthesize antibody-drug conjugates by using thiols, amines, alcohols, aldehydes and azides.

## 1. Introduction

Cancer is still one of the major threats to human health. However, cancer therapies used today always have more or less adverse side effects to normal tissues. Targeted therapy is a promising strategy to address this challenge. The pioneer of targeted therapy is Paul Ehrlich who introduced the principle “magic bullet” at the beginning of the 20th century [1]. To avoid side effects, drugs should be specifically delivered to cancer cells via binding to ligands that can specifically recognize the cancer-associated biomarkers such as antigens. Among the ligands for targeted therapy, antibodies are excellent candidates because of their specific recognitions and high affinities. Nowadays, antibody-drug conjugates (ADCs) are attracting tremendous attention for targeted cancer therapy.

**Figure 1:**
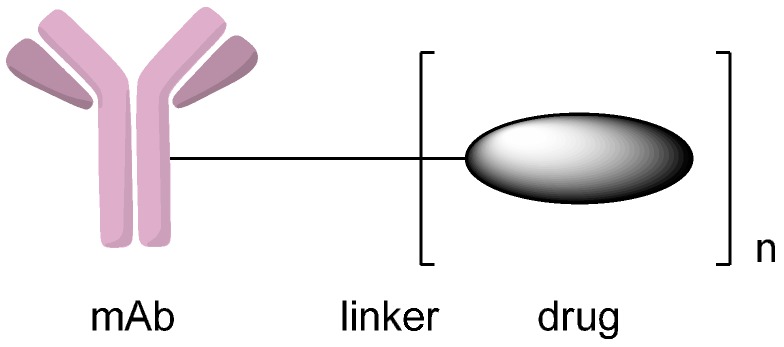
Schematic representation of an antibody-drug conjugate (ADC). Reprinted with permission from Reference [2].

Antibody-drug conjugates are biotherapeutics that consist of monoclonal antibodies, potent cytotoxic drugs and linkers between them (Figure 1). The monoclonal antibodies lead the drug precursors to the target cancer cells, in which the prodrugs can be chemically or enzymatically converted to drugs in their active forms [2]. Conjugating cytotoxins to monoclonal antibodies that specifically tie to tumor cell surface antigens enables the drugs to be target-delivered to cancer cells and leaves normal cells unaffected. More important, many of the cytotoxic drugs that are too toxic for use in traditional chemotherapy can also be used in the construction of antibody-drug conjugates [3,4]. The linkers are also essential parts of antibody-drug conjugates, which account for stability in circulation, good pharmacokinetics and efficient release of toxic drugs in the tumor cells.

The selection of antibody, drug, and linker has recently been summarized in a few excellent reviews [5,6,7,8,9,10,11]. In this review, we mainly describe the linking methods to design and synthesize ADCs, including those that are not discussed in the reviews mentioned above.

## 2. Conjugation via Various Functional Groups to Synthesize Antibody-Drug Conjugates (ADCs)

### 2.1. Conjugation via Thiols

Employing the thiols of interchain cysteine residues in monoclonal antibodies as attachment sites for drug molecules is one of the most used conjugation methods. In a human IgG1, there are four interchain disulfide bonds that can be used as potential conjugation sites [12]. The four interchain disulfide bonds can be reduced by tris(2-carboxyethyl) phosphine (TCEP) or dithiothreitol (DTT), which results in eight thiol groups that are available for conjugating drug molecules. Through this method, different drug antibody ratio (DAR) conjugates will be obtained when targeting typical DARs of 2–4 [13,14]. In addition, antibody-drug conjugate at each drug antibody ratio has several isomers. Thus, over a hundred different species are present in the antibody-drug conjugate. Although conventional methods that employ cysteine residues as conjugation sites are highly heterogeneous, Adcetris^®^ was approved by FDA in 2011.

**Scheme 1:**
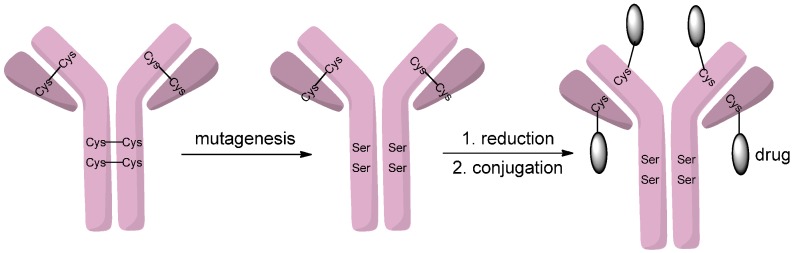
Interchain cysteine to serine mutagenesis enables drugs to conjugate to the remaining cysteines. Adapted from reference [18].

Homogeneous antibody-drug conjugates can be produced through cysteine residues when all interchain cysteines are coupled to drugs. For example, Senter and coworkers [15,16] developed such a conjugate which consisted of cAC10, an anti-CD30 monoclonal antibody, and monomethyl auristatin E (MMAE). This cAC10-vcMMAE conjugate contains eight drugs per antibody, which is the highest drug antibody ratio (DAR) that can be obtained through using interchain cysteines as conjugation sites. However, antibody-drug conjugates with four drugs per antibody generally have improved *in vivo* performance [17]. McDonagh *et al.* [18] developed a method to control the conjugate sites by mutating four or six of the interchain cysteines to serines, therefore leaving four or two cysteines accessible for conjugating (Scheme 1). After reduction of the disulfide bonds, the mutated monoclonal antibodies with the reduced number of interchain cysteines were conjugated with the drug vcMMAE. Through this method, homogenous antibody-drug conjugates with clear attachment sites could be produced.

Reducing the disulfide bonds of a monoclonal antibody should not affect its functions [19]. What is more, interchain disulfide bonds are easier to be reduced than intrachain disulfide bonds [20]. These allow free thiol groups to be generated under mild reducing conditions while leaving the antibody intact at the same time. Liu *et al.* [21] took advantage of the fact that different disulfide bonds in a monoclonal antibody have different susceptibilities towards reduction and developed another strategy to tightly control the site of conjugation. Limited reduction with TCEP or DTT predominantly yielded conjugates in which drugs were attached to heavy-light chain disulfides; partial re-oxidation of fully reduced antibodies with 5,5′-dithiobis (2-nitrobenzoic acid) (DTNB) yielded conjugates that drugs were mainly attached to by heavy-heavy chain disulfides [13].

#### 2.1.1. Addition to Maleimides

**Scheme 2:**
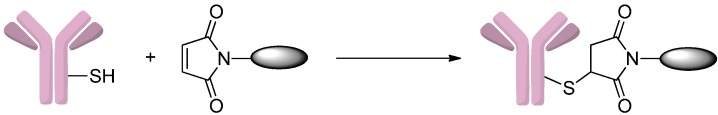
The synthesis of antibody-drug conjugates (ADCs) through the addition of thiols to maleimides. Adapted from reference [23].

Classically, cysteine residues can be modified through addition of thiols to electrophiles such as maleimides (Scheme 2) [22,23,24,25]. The conjugate could be achieved by reducing the disulfide bonds of the antibody and then adding to maleimides. Addition to maleimides is the most common method for attaching drugs to antibodies. Adcetris^®^, which was approved by the FDA for the treatment of patients with Hodgkin’s lymphoma after failed autologous stem cell transplantation or patients with systemic anaplastic large-cell lymphoma after the failure of at least one prior multi-agent chemotherapy regimen, was produced by this method in which a maleimide-functionalized drug was conjugated to the interchain cysteine residues of an anti-CD30 antibody [15]. Maleimide-based antibody-drug conjugates were recently found to have limited stability in blood circulation [26], which would lower the efficacy of the conjugates and damage healthy tissue. Succinimide or maleimide hydrolysis is a promising method to get around this problem. Once hydrolyzed, the antibody-drug conjugates were no longer subject to elimination reactions of maleimides through retro-Michael reactions, thus improving the stabilities and potencies of ADCs [27,28,29].

#### 2.1.2. Disulfide-Thiol Exchange

**Scheme 3:**
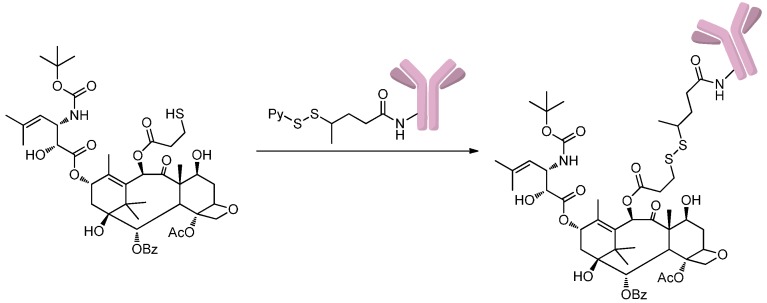
Preparation of antibody-taxoid conjugates via disulfide-thiol exchange. Adapted from reference [30].

The approach disulfide-thiol exchange could also be used to synthesis ADCs by forming a new disulfide bond between drugs and antibodies [30,31]. Ojima *et al.* [30] designed and synthesized novel antibody-taxoid conjugates that include highly cytotoxic taxoid drug and monoclonal antibodies that could recognize the EGFR expressed in cancer cells. In this study, taxoid bearing a free thiol group was attached to the pyridyldithio groups of the modified anti-EGFR antibodies through disulfide-thiol exchange (Scheme 3). The resulting conjugates possess remarkable antitumor activities against EGFR-expressing A431 (human epidermoid) tumor xenografts in immune deficient mice.

#### 2.1.3. Addition to Alkynes

**Scheme 4:**
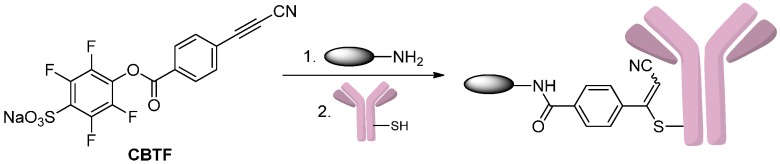
The conjugation of amine-drug to trastuzumab by using sodium 4-((4-(cyanoethynyl)benzoyl)oxy)-2,3,5,6-tetrafluorobenzenesulfonate (CBTF). Reprinted with permission from reference [32].

To avoid the maleimide instability issue, Kolodych *et al.* [32] developed a heterobifunctional reagent, sodium 4-((4-(cyanoethynyl)benzoyl)oxy)-2,3,5,6-tetrafluorobenzenesulfonate (CBTF), for amine-to-thiol coupling (Scheme 4). This reagent comprises a 3-arylpropionitrile (APN) group that replaces the maleimide and allows for the preparation of remarkably stable conjugates. Addition of thiols in the antibodies to the 3-arylpropionitriles predominantly produced *Z*-isomers of the addition products.

#### 2.1.4. Disulfide Re-Bridging

**Scheme 5:**
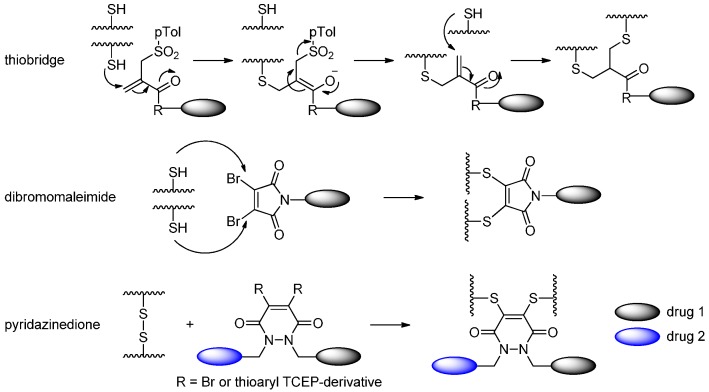
Three approaches to make ADCs through disulfide re-bridging: thiobridge, dibromomaleimide and pyridazinedione. Adapted from reference [35,37,40].

Recently, various novel cysteine-relied conjugation methods have been developed [33]. Godwin and coworkers [34,35,36] developed a thiol conjugation approach in which interchain disulfide bonds of the cysteines were partially reduced, followed by bis-alkylation (including Michael addition and elimination) to introduce thiols of two cysteines to the drug (Scheme 5). Depending on the reduction degree, the numbers of cysteines for conjugation can be eight or four to generate drug antibody ratios (DARs) of four and two, respectively. They also demonstrated that the thiobridge ADCs are more stable than maleimide ADCs in the human serum.

Behrens *et al.* [37] reduced all the disulfide bonds, exposing eight cysteine residues, then similarly used dibromomaleimide (DBM) to react with the free thiol groups of the antibody and produced a dithiomaleimide (DTM) ADC. Four cytotoxic drugs with this functional linker were attached to the monoclonal antibodies conveniently by linking with the cysteine residues.

Chudasama and coworkers [27,38,39,40] presented a significant method towards next-generation antibody-based therapeutics through disulfide re-bridging. In their works, the reduction of disulfides and disulfide re-bridging could be achieved in one step by the use of a single reagent: dithioaryl(TCEP)pyridazinedione [38]. Disulfide re-bridging through the use of dibromopyridazinedione derivatives after disulfide reduction by TCEP was another strategy for the construction of antibody-based therapeutics in their studies [39,40]. The resulting conjugates were highly stable and had potent cytotoxicites against tumor cells.

#### 2.1.5. Expressed Protein Ligation (EPL) and Alkylguanine-DNA-Alkyl Transferase (AGT) Reaction

**Scheme 6:**
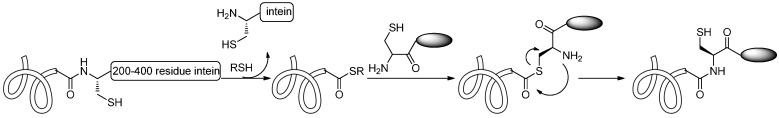
Amino mercapto-derivitized drug can be attached to an antibody through intein splicing. Reprinted with permission from reference [34].

Another strategy for making homogeneous ADCs was inserting entire domains or proteins into antibodies. The most well known such method is expressed protein ligation (EPL), which relied on a self-splicing intein to activate the C-terminal of the target protein and thus formed a new amide bond with a small molecule, peptide or protein. EPL followed the mechanism: (1) C-terminal thioester formation through the spontaneous N to S rearrangement of an intein; (2) selected thiol displacement of the intein sequence to give an activated thioester; (3) thiol exchange of the thioester with a β-amino mercapto ligand (small molecule, peptide or protein); and (4) spontaneous N to C rearrangement to form an stable amide bond that links the antibody of interest to the drug with a inserted cysteine (Scheme 6) [34].

**Scheme 7:**
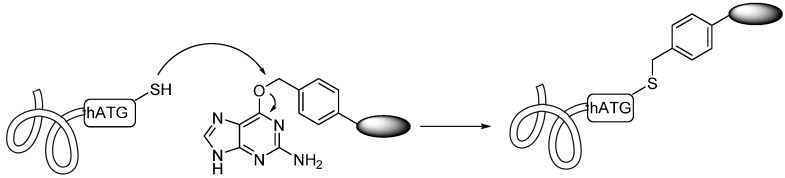
Human O^6^-alkylguanine-DNA alkyltransferase (hAGT) used guanine as a leaving group, forming a thioether bond to a benzyl-derivitized drug. Adapted from Reference [41].

Proteins that do not interfere with the function of an insertion can take advantage of the human O^6^-alkylguanine-DNA alkyltransferase (hAGT) reaction in which the guanine attached to the O^6^ benzyl group is attacked by the cysteine of hAGT and thus transferred to the drug-AGT conjugate (Scheme 7). To realize this reaction, hAGT was directly evolved to possess comparable kinetics to the wild type hAGT, while retaining the substrate tolerance for the *O*-benzyl moiety [41].

### 2.2. Conjugation via Amines

#### 2.2.1. Formation of Amides

Forming amide is one of the most important reactions for the nucleophilic amines. Amines could typically be acylated by carboxyl via some familiar activating reagents, such as *N*-hydroxysuccinimide (NHS), 2-Succinimido-1,1,3,3-tetra-methyluronium tetrafluoroborate (TSTU), and Benzotriazol-1-yl-oxytripyrrolidinophosphonium hexafluorophosphate (PyBOP).

**Scheme 8:**
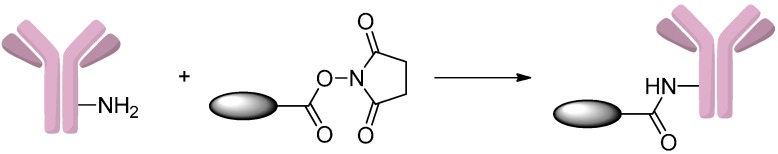
Amines of the antibodies reacted with the carboxyls that derived from the drugs in the effect of the *N*-hydroxysuccinimide (NHS). Adapted from reference [23].

Amines of the antibodies can react with the carboxyls that derived from the drugs in the effect of the NHS to give antibody-drug conjugates (Scheme 8) [22,23,42]. Amines of lysines are commonly used for linking drugs to antibodies because lysines are usually exposed on the surface of the antibodies and therefore easily accessible. Antibodies contain up to 80 lysines [43] and, as a result, conjugation through lysine residues inevitably leads to twofold heterogeneity: (1) different number of drugs per antibody; and (2) antibodies with the same number of drugs attached at different sites [31,44]. The heterogeneity with respect to DARs can be restricted to a certain extent by adjusting the stoichiometry of drug and antibody used in the reaction; and with respect to site-specificity, the heterogeneity can be limited by the chemical accessibility of reactive groups [45,46]. Mylotarg^®^ was the first antibody-drug conjugate on the market by lysine-coupling. In the conjugate, a semi-synthetic calicheamicin derivative was activated with NHS, and then attached to the lysines of a humanized IgG4 [47]. However, Mylotarg^®^ was withdrawn from the market in 2010 due to the lack of benefit improvement to patients. Recently, there was a new clinically relevant antibody-drug conjugate generated by lysine modification: Kadcyla^®^ [48].

Hong *et al.* [49] developed an approach to covalently attach the anticancer drug doxorubicin to an anti-EGFR antibody fragment (Fab’) through a polyethylene glycol (PEG) linker. In this work, CIT–(CH_2_)_5_–PEG_24_–CO_2_H was activated by TSTU or PyBOP and then coupled *in situ* with the C3′-amine of DOX to give CIT–(CH_2_)_5_–PEG_24_–DOX, which was then conjugated with the cysteine residues of an anti-EGFR Fab’. Introduction of PEG increased aqueous solubility of the drug, which led to a yield improvement of the conjugation reaction with the Fab’.

**Scheme 9:**
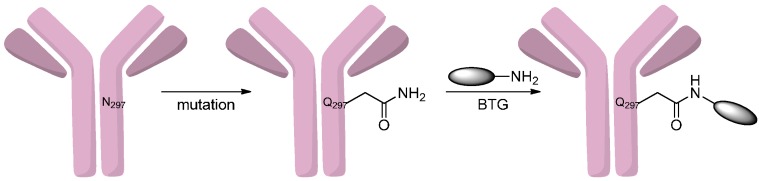
The reaction of the amine-drugs and antibodies under the influence of bacterial transglutaminase (BTG). Adapted from reference [50].

Besides familiar activating reagent, amines could react with carboxyl acids and their derivatives to form the amides under the influence of the BTG, SrtA and BirA. For example, Jeger *et al.* [50] observed the selective acylation of amines by the glutamines in the heavy chain’s flexible regions of an IgG where the asparagines (N) were mutated to glutamines (Q). By using bacterial transglutaminase (BTG) at these sites, they synthesized homogeneous conjugates that were tumor-uptake selective *in vivo* (Scheme 9).

**Scheme 10:**
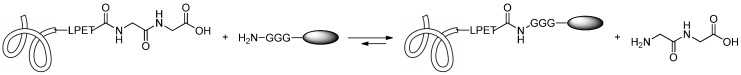
The sortase-mediated conjugation of glycine-derivitized drugs and the antibodies. Adapted from reference [54].

Sortase A (SrtA), one of the many sortases found in Gram-positive bacteria, is a transpeptidase [51] that can recognize a LPXTG sequence, break the TG bond and facilitate the formation of a new amide bond with the amine of glycine-derivitized drug. This was demonstrated through conjugating folate, biotin, rhodamine and so on [52,53]. The leaving glycine could return as a competing nucleophile, which reversed the reaction, unless more than 10 equivalents of glycine-derivitized drugs were used (Scheme 10) [54]. Williamson *et al.* [55] put forward a creative solution to solve this problem: they used threonine esters as substrates and the product glycolic acids were far less nucleophilic, enabling conjugation with a 1:1 stoichiometry.

**Scheme 11:**
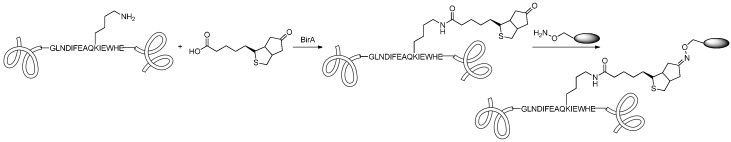
The BirA-mediated conjugation of biotin-like ketones, oxyamine-drugs and antibody. Adapted from Reference [56].

The biotin ligase (BirA) could recognize and acylate the Avi-tag, which is a 15-amino acid GLNDIFEAQKIEWHE sequence. Chen *et al.* [56] found that BirA could accept keto analogs of biotin as substrates to react with Avi-tag and form intermediates that could conjugate with the oxyamines of drugs (Scheme 11). BirA has a similar function as formylglycine-generating enzyme (FGE), which can site-specifically insert an aldehyde group into an antibody. In this case, a keto group was introduced into the antibody. Although BirA requires a long sequence, it is far less restricted in the location along the antibody. They demonstrated the reaction with the installed carbonyl using fluorescein hydrazide.

#### 2.2.2. Formation of Carbamates

**Scheme 12:**
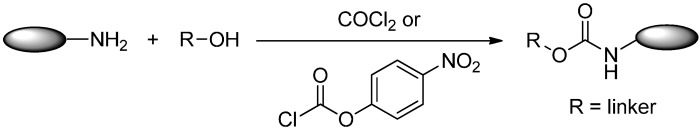
The reaction of amines of the drug and hydroxyls of the linkers in the effect of phosgene or 4-nitrophenyl chloroformate. Adapted from Reference [57,58].

Amines of the drugs could react with the hydroxyls that derived from the linkers in the effect of phosgene, 4-nitrophenyl chloroformate, *etc.* and form the carbamate containing drug-linkers (Scheme 12), which was then coupled to antibodies [57,58]. For instance, Jeffrey *et al.* [57] prepared antibody-drug conjugates in which the amino-CBI drug, a DNA minor groove binder drug (MGBs), was attached to monoclonal antibodies through peptide linkers that designed to release drugs in the lysosomes of target cells. In this study, the amino-CBI drug reacted with phosgene to form the corresponding isocyanate and then the linker with a hydroxyl was added to form the carbamate. Dubowchik *et al.* [59] linked the anticancer drug doxorubicin to chimeric BR96, an internalizing monoclonal antibody, through lysosomally cleavable dipeptides. In this case, the carbamate between drug and antibody was prepared with 4-nitrophenyl chloroformate. These antibody-drug conjugates usually insert a spacer such as *para*-aminobenzyl carbamate (PABC) between the peptide linkers and the drugs to minimize the steric interaction effects. This approach has previously been used to release doxorubicin [60], MMAE [17] and camptothecin [61] from antibody-drug conjugates.

Another carbamate was designed for the hydroxy containing drug. For instance, antibody-drug conjugate SYD985 consists of *seco*-DUBA drug, self-elimination spacer, cleavable peptides linker and trastuzumab. The *seco*-DUBA drug was linked to the self-elimination spacer via carbamate bond that derived from carbonate. Treatment of MOM protected duocarmycin with 4-nitrophenyl chloroformate gave the corresponding carbonate. Commercially available *tert*-butyl methyl(2-(methylamino) ethyl)carbamate was then used to synthesize the carbamate. Removal of the Boc and MOM groups with trifluoroacetic acid (TFA) provided cyclization spacer-duocarmycin as a TFA salt. Cyclization spacer-duocarmycin was reacted with the activated linker to synthesis drug-linker module under slightly basic conditions [62].

### 2.3. Conjugation via Alcohols

#### 2.3.1. Formation of Carbonates

Similar with amines, alcohols can react with chloroformates to form carbonates. For example, Moon *et al.* [63] conjugated 7-ethyl-10-hydroxycamptothecin (SN-38) derivatives to hMN-14, a humanized anti-CEACAM5 mAb, via a carbonate bond. To construct the carbonate bond, BOC-SN-38 [64] was firstly converted to its 20-*O*-chloroformate, and then reacted *in situ* with the known linker, MC-Phe-Lys(MMT)-PABOH [60]. 

#### 2.3.2. Formation of Ether Bonds

**Scheme 13:**
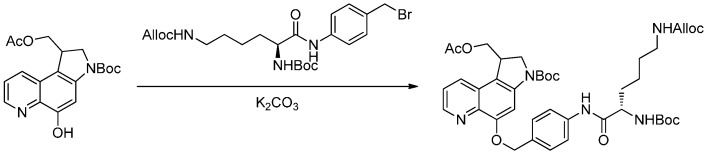
The formation of an ether bond through the *O*-alkylation of aza-CBI by bromide and potassium carbonate. Adapted from reference [57].

Jeffrey *et al.* [57] described a method to conjugate amino-CBI drug to the monoclonal antibody by formation of carbamates (see Section 2.2.2). Another approach for attaching a DNA minor groove binder drug (MGB) derivative to mAb involved *O*-alkylation of the hydroxy in aza-CBI to form ether bond. In this approach, a *para*-aminobenzyl ether (PABE) group was used as a self- elimination spacer between the drug and the peptides [57]. An important step in the synthesis of this antibody-MGB conjugate was the formation of an ether bond through the *O*-alkylation of aza-CBI by bromide and potassium carbonate (Scheme 13). This work also showed that the conjugate could cleave via amide bond hydrolysis and lead to the release of free phenolic drug [65]. This approach should be broadly applied to drugs that have a phenolic hydroxyl group as the conjugate site [66].

#### 2.3.3. Formation of Ester Bond

In 2010, Quiles *et al.* [67] developed paclitaxel-monoclonal antibody (PTX-mAb) conjugates that could deliver significant doses of drugs to the tumor cells using the ester bond. The conjugates used PEG as linker, and the paclitaxel was attached to the linker with glutarate (GL) or succinate (SX) through the ester bond, the resulting PTX–L–Lys[(PEG_12_)_3_–PEG_4_]–PEG_6_–CO_2_NHS (L = GL or SX) was then conjugated to C225, an antiepidermal growth factor receptor (anti-EGFR) monoclonal antibody, producing completely soluble conjugates.

### 2.4. Conjugation via Aldehydes

#### 2.4.1. Conjugation via FGE

**Scheme 14:**
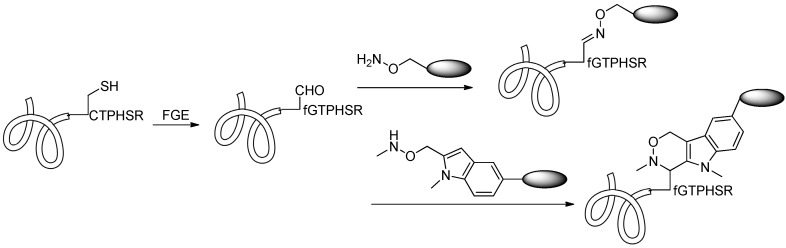
The conversion of the cysteine thiol to an aldehyde group by formylglycine-generating enzymes (FGE) enables reactions with oxyamine drugs. Adapted from reference [70].

Conjugation via aldehydes is another method for linking drugs to antibodies. Formylglycine-generating enzymes (FGEs), which recognize and modify a short CXPXR (where X is any amino acid) sequence, can be used to modify the cysteine residues of antibodies to aldehyde-containing formylglycine (FGly) residues. This method was applied to generate site-specific antibody-drug conjugates via incorporating cytotoxic drugs into monoclonal antibodies with a formylglycine [68]. Following the production of modified antibody, a chemical method can be used to conjugate a drug to the aldehyde group of formylglycine. Oxyamine or hydrazide drugs were attached to the modified antibodies successfully (Scheme 14) [69]. Recently, this kind of aldehyde conjugation strategy was further developed by Agarwal *et al.* [70], who used hydrazino-iso-Pictet-Spengler (HIPS) chemistry to attach maytansine to the aldehyde-containing trastuzumab. The HIPS chemistry resulted in the formation of a covalent C–C bond, which was more stable than oxime or hydrazone ligation products in physiological condition. What is more, this study showed that the aldehyde group can be introduced in many locations of the antibody without affecting the stability and activity of the obtained antibody-drug conjugates.

#### 2.4.2. Conjugation via aaRS

**Scheme 15:**
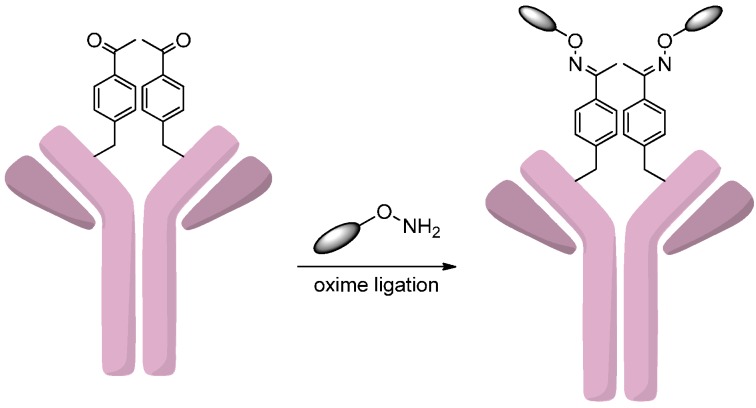
Incorporation of *p*-acetylphenylalanine (pAcPhe) allows for site-specific conjugation of drugs to the modified antibodies. Adapted from reference [74].

The tRNA/aminoacyl-tRNA synthetase (aaRS) pair can site-specifically incorporate an unnatural amino acid (e.g., *p*-acetylphenylalanine, pAcPhe) into antibody [71]. Recently, the genetic incorporation of unnatural amino acids into antibodies had become a useful tool in the ADC design [72,73]. This method was successfully realized by Axup *et al.* [74], who developed site-specific auristatin conjugates of trastuzumab. A *p*-acetylphenylalanine was loaded onto the amber codon of tRNA by aaRS and then specifically incorporated into the amber site of the trastuzumab heavy chain. After purification, the antibody was coupled to the monomethyl auristatin F (MMAF) derivative that contains an oxyamine group by an oxime ligation with the pAcPhe residues (Scheme 15). The analysis of antitumor activity and pharmacokinetics of this site-specific antibody-drug conjugate confirmed its efficacy and stability in serum.

#### 2.4.3. Oxidation of Sialic Acids

**Scheme 16:**
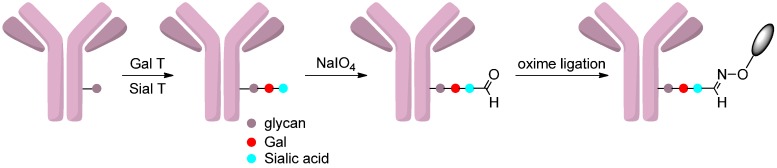
The modification of glycans of trastuzumab by Gal T and Sial T leads to the incorporation of sialic acids, which were oxidated and coupled to the drugs through oxime ligation. Adapted from reference [75].

Glycoengineering has been employed to synthesize site-specific antibody-drug conjugates, in which sialic acids were used as chemical handles for selective conjugations. This was achieved by incorporating sialic acids into the native glycans of trastuzumab through β-1,4-galactosyltransferase (Gal T) and α-2,6-sialyltransferase (Sial T). Prior to reaction with the oxyamine drugs, the alcohol groups of sialic acids were oxidated to aldehyde groups. The resulting antibodies could react with the cytotoxic drugs via an oxime ligation (Scheme 16). This method was evaluated by conjugating trastuzumab with two drugs, monomethyl auristatin E (MMAE) and dolastatin 10. The glycoengineered antibody-drug conjugates exhibited comparable antitumor activities to the conventional analogs [75].

#### 2.4.4. Conjugation via Transamination Reagent

**Scheme 17:**
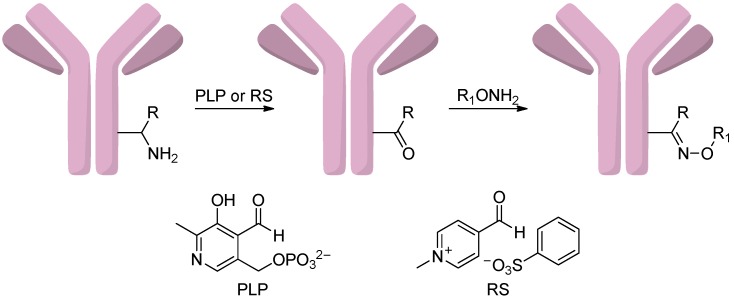
The oxidation of amines to aldehyde groups which could react with oxyaminedrugs using transamination reagents pyridoxal 5′-phosphate (PLP) or *N*-methylpyridinium-4-carboxaldehyde benzenesulfonate salt (RS). Adapted from reference [79].

Witus *et al.* [76,77] introduced carbonyl groups by transamination reagent pyridoxal 5′-phosphate (PLP) at the N-terminus of antibodies, which could be used as unique attachment sites for the conjugation formation (Scheme 17). However, the reaction yields were not very high and high temperatures were required, which limited the application of this method. To solve these problems, they developed a combinatorial peptide library screening platform and found a new transamination reagent, *N*-methylpyridinium-4-carboxaldehyde benzenesulfonate salt (RS) [78]. Antibodies with glutamate-rich sequences were particularly reactive substrates for this reagent [79].

### 2.5. Conjugation via Azides

#### 2.5.1. Click Reactions with DBCO

**Scheme 18:**
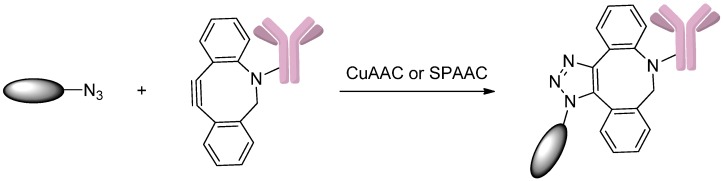
The synthesis of ADCs through copper-catalyzed azide-alkyne cycloaddition (CuAAC) and strain-promoted azide-alkyne cycloaddition (SPAAC) click reactions. Adapted from reference [81].

Azides can react with alkynes to form triazoles through click chemistries, such as copper-catalyzed azide-alkyne cycloaddition (CuAAC) and strain-promoted azide-alkyne cycloaddition (SPAAC) (Scheme 18) [80,81]. This approach was recently used to construct antibody-drug conjugates. For example, Zhou *et al.* [81] conjugated drugs to antibodies using this method. In this study, an azide-containing reagent, sodium (difluoroalkylazido)sulfinate (DAAS-Na), allowed azide groups to be linked to heteroaromatics, and the products could then be attached to monoclonal antibodies by click reactions. DAAS-Na was used in the heteroarene functionalization reaction, in which ZnCl_2_ and TsOH·H_2_O were acid additives and tBuOOH was an oxidant. The resulting azide-linked drugs could react with a dibenzylcyclooctyne (DBCO) containing antibody through a CuAAC reaction. This strategy expands the extent of bioactive drugs that can be linked to monoclonal antibodies.

Microbial transglutaminase (MTGase) can catalyze the formation of an isopeptide bond between the amine group of glutamine and the primary amine of lysine while simultaneously releasing an ammonia gas [82]. The coupling activity of MTGase was applied to synthesize antibody-drug conjugates [83,84,85]. For example, Dennler *et al.* [83] afforded a highly homogeneous trastuzumab-MMAE conjugate with DAR of 2 using this enzymatic conjugation strategy. In this work, an azide-containing linker, which involves a primary amine, was coupled to Q295 of the deglycosylated antibody by MTGase. This enzymatic reaction was followed by a SPAAC reaction with the DBCO-containing auristatin drug.

Cell-free protein synthesis (CFPS) system was also efficiently applied to produce monoclonal antibodies that contain unnatural amino acids for antibody-drug conjugate generations. For example, Zimmerman *et al.* [86] prepared a site-specific antibody-drug conjugate via CFPS system using a new synthetase to incorporate a *para*-azidomethylphenylalanine (pAMF) to the monoclonal antibody, which was then linked to a DBCO-functionalized MMAF drug by SPAAC reaction.

#### 2.5.2. Click Reactions with Terminal Alkynes

**Scheme 19:**
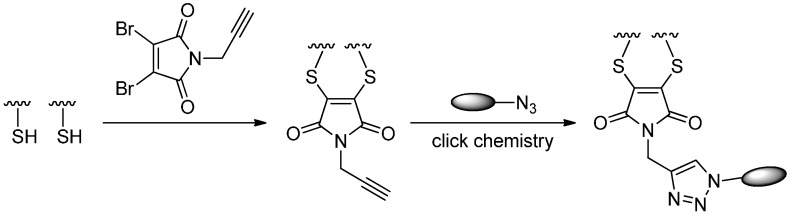
The synthesis of trastuzumab-porphyrin conjugates through *N*-propargyl-3,4-dibromomaleimide and a click chemistry. Reprinted with permission from Reference [87].

In 2014, Bryden *et al.* [87] described the attachment of azide-functionalized porphyrins to a tratuzumab via a novel conjugation method. In this study, Trastuzumab was treated with TCEP in order to reduce the interchain disulfide bond. Treatment with *N*-propargyl-3,4-dibromomaleimide yielded alkyne-containing trastuzumab, which then successfully reacted with porphyrin derivatives through the CuAAC reaction to afford trastuzumab-porphyrin conjugates (Scheme 19). This method could also be realized using SPAAC reaction [39].

## 3. Conclusions

A promising approach to improve the potency of drugs is to be conjugated to monoclonal antibodies that enable these cytotoxic drugs to be site-specifically delivered to tumor cells while avoiding the toxicity of drugs on normal cells. The linkers of antibody-drug conjugates profoundly impact their potency and safety. Recently, a variety of methods have been developed to conjugate drugs to antibodies. In this review, we summarized the methods that are currently used to design and synthesize antibody-drug conjugates, including heterogeneous ADCs and homogeneous ADCs, via various functional groups such as thiols, amines, alcohols, aldehydes and azides. Heterogeneous ADCs were usually synthesized through the thiols of cysteine residues and the amines of lysines, however, the heterogeneity diminished their activities and promoted antibody aggregations, and increased toxicities in the circulation. Homogeneous ADCs made through the catalysis of site-specific conjugation enzymes such as AGT, BTG, aaRS and Sial T are more stable and have comparable or even better activities than those conventional analogs *in vivo*. We believe that a growing number of methods will be developed to synthesize ADCs in the near future, and more and more ADCs, especially site-specifically modified ADCs, will be produced.
